# Supplementary material for: Lymph Vessels Associate with Cancer Stem Cells from Initiation to Malignant Stages of Squamous Cell Carcinoma
Source: Int J Mol Sci. 2023 Sep 2;24(17):13615. doi: 10.3390/ijms241713615 (PMC10488284; doi:10.3390/ijms241713615)
Supplement: Supplementary file 1 [file ijms-24-13615-s001.zip › Supplementary Information IJMS_v7.pdf]

SUPPLEMENTARY MATERIALS for

Lymph vessels associate with cancer stem cells from initiation to malignant stages of squamous cell carcinoma

Anna Cazzola [1], David Calzón Lozano [1], Dennis Hirsch Menne [1], Raquel Davila Pedrera [1], Jingcheng Liu [1], Daniel Peña-Jimenez [2], Silvia Fontenete [1], Cornelia Halin [3], Mirna Perez-Moreno [1*]

1 Section for Cell Biology and Physiology. Department of Biology, University of Copenhagen, Copenhagen, 2100, Denmark.

2 Unidad de Investigación Biomédica, Universidad Alfonso X el Sabio (UAX), Avenida de la Universidad 1, Villanueva de la Cañada, Madrid, 28691, Spain.

3 Institute of Pharmaceutical Sciences, ETH Zurich, Zurich, 8093, Switzerland.

*Correspondence: mirna.pmoreno@bio.ku.dk

Supplementary Figures and Supplementary Figure Legends

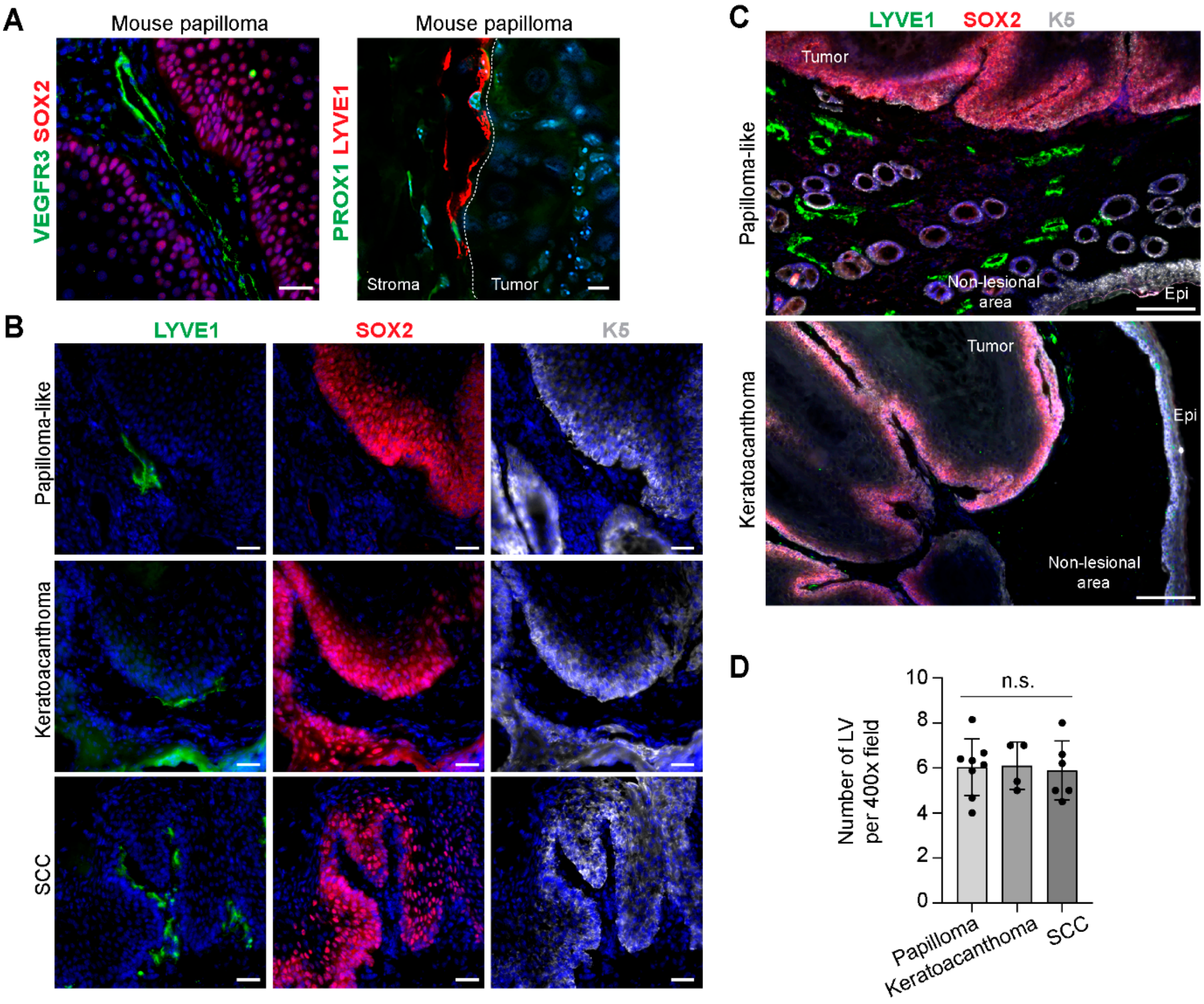

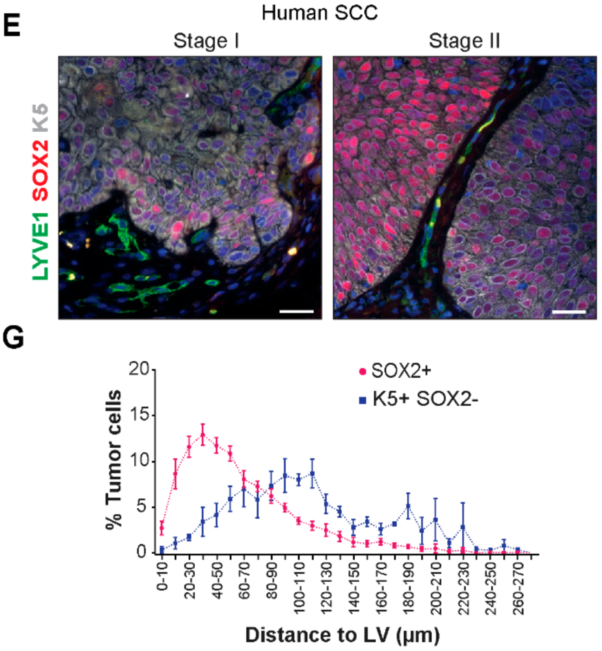

F

Sample	SCC location	TNM	Grade	Stage	Age	Gender
1	leg	T1N0M0	1	I	73	M
2	back	T2N0M0	1	II	58	M
3	face	T2N0M0	1	II	90	F
4	leg	T1N0M0	1	I	73	M
5	limb	T2N0M0	1	II	56	M
6	foot	T1N0M0	2	I	66	M
7	post aurem	T2N0M0	2	II	64	M
8	NA	T1N0M0	1	I	NA	NA
9	NA	T1N0M0	2	I	NA	NA
10	NA	T1N0M0	1	I	NA	NA

Figure S1. LV distribute at the CSC niche in human skin squamous cell carcinoma at different stages. (**A**) Representative immunostaining of mouse skin papillomas for the LV marker VEGFR3 (green) and the CSC marker SOX2 (red) and for the LV markers PROX1 (green) and LYVE1 (red). Hoechst nuclear staining is shown in blue. The dotted line indicates the tumor stroma border. Scale bars=30 µm. (**B**) Mouse skin immunostaining for the LV marker LYVE1 (green), the CSC marker SOX2 (red), and the basal progenitor keratinocyte marker K5 (white). Hoechst nuclear staining is shown in blue. Scale bar=30 µm. (**C**) Surrounding perilesional regions immunostaining for the LV marker LYVE1 (green), the CSC marker SOX2 (red), and the basal progenitor keratinocyte marker K5 (white). Hoechst nuclear staining is shown in blue. Epi. Epidermis. Scale bar=250 µm. (**D**) Histogram of the number of $LYVE1^{+}$ LV per 400x field of skin tumor lesions. $n \geq 4$, n.s. non-significant $p>0.05$. (**E**) Human skin SCC immunostaining for the LV marker LYVE1 (green), the CSC marker SOX2 (red), and K5 (white). Hoechst nuclear staining is shown in blue. Scale bars=30 µm. (**F**) Clinical parameters of sSCC specimens and patients analyzed in the study. (**G**) Distribution of SOX2+ CSC relative to the distance to LV, n=10 SCCs, and distribution of K5+ SOX2- tumor cells relative to the distance to LV, n=4 SCCs.

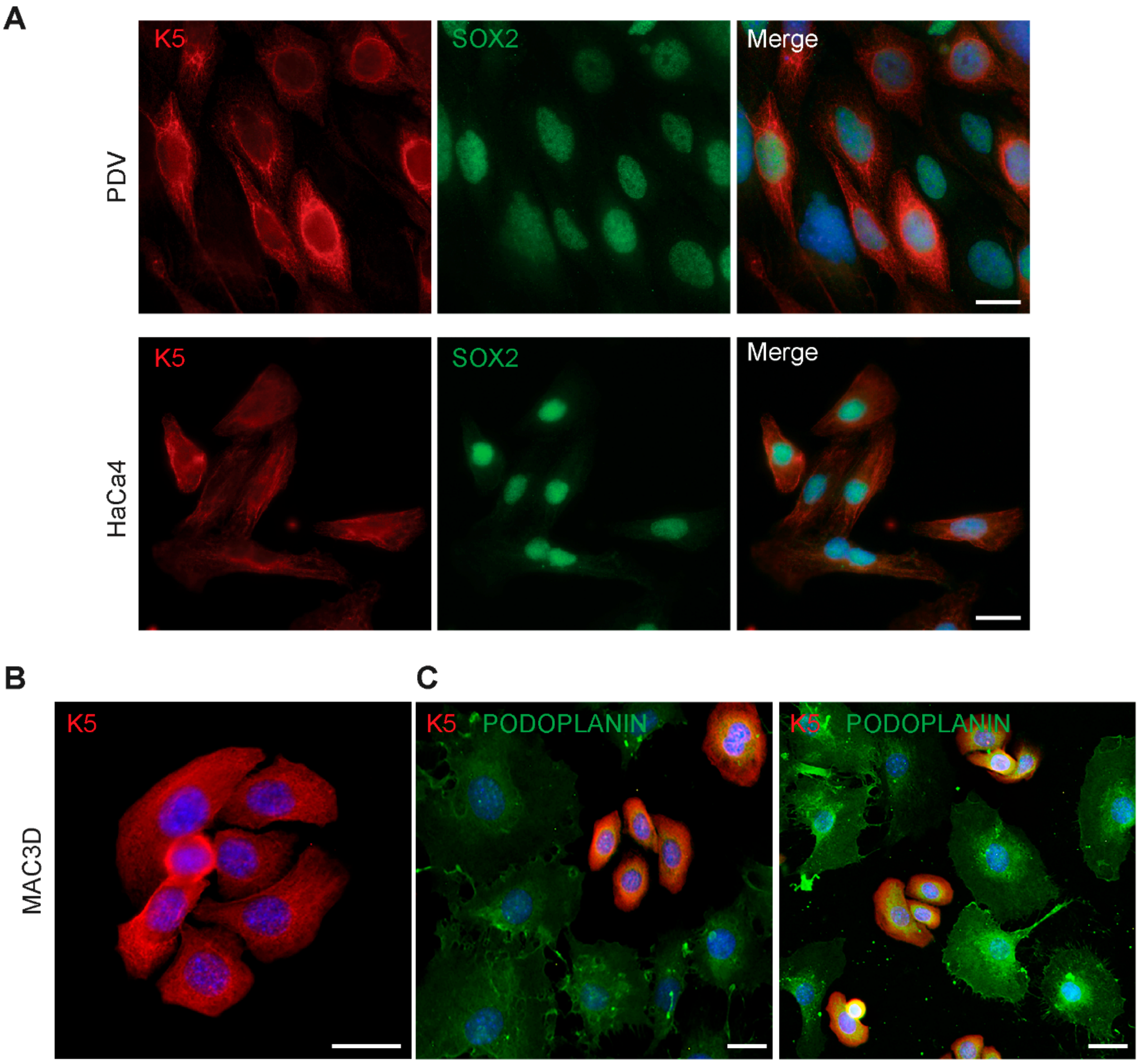

Figure S2. The cancer cell lines PDV and HaCa4 express the CSC marker SOX2. (**A**) Immunofluorescence staining of the cancer cells PDV and HaCa4 for K5 (red) and the CSC marker SOX2 (green). Hoechst nuclear staining is shown in blue. Scale bar=20 µm. (**B**) Immunofluorescence staining of the immortalized mouse keratinocyte cell line MCA3D for K5 (red). Hoechst nuclear staining is shown in blue. Scale bar=20 µm. (**C**) Two representative immunofluorescence images of co-cultured MCA3D (K5, red; PODOPLANIN, green) and LEC (PODOPLANIN, green), showing the lack of heterotypic interactions and the differential segregation of the two cell types (n=2, 3 technical replicates each experiment). Hoechst nuclear staining is shown in blue. Scale bar=20 µm.

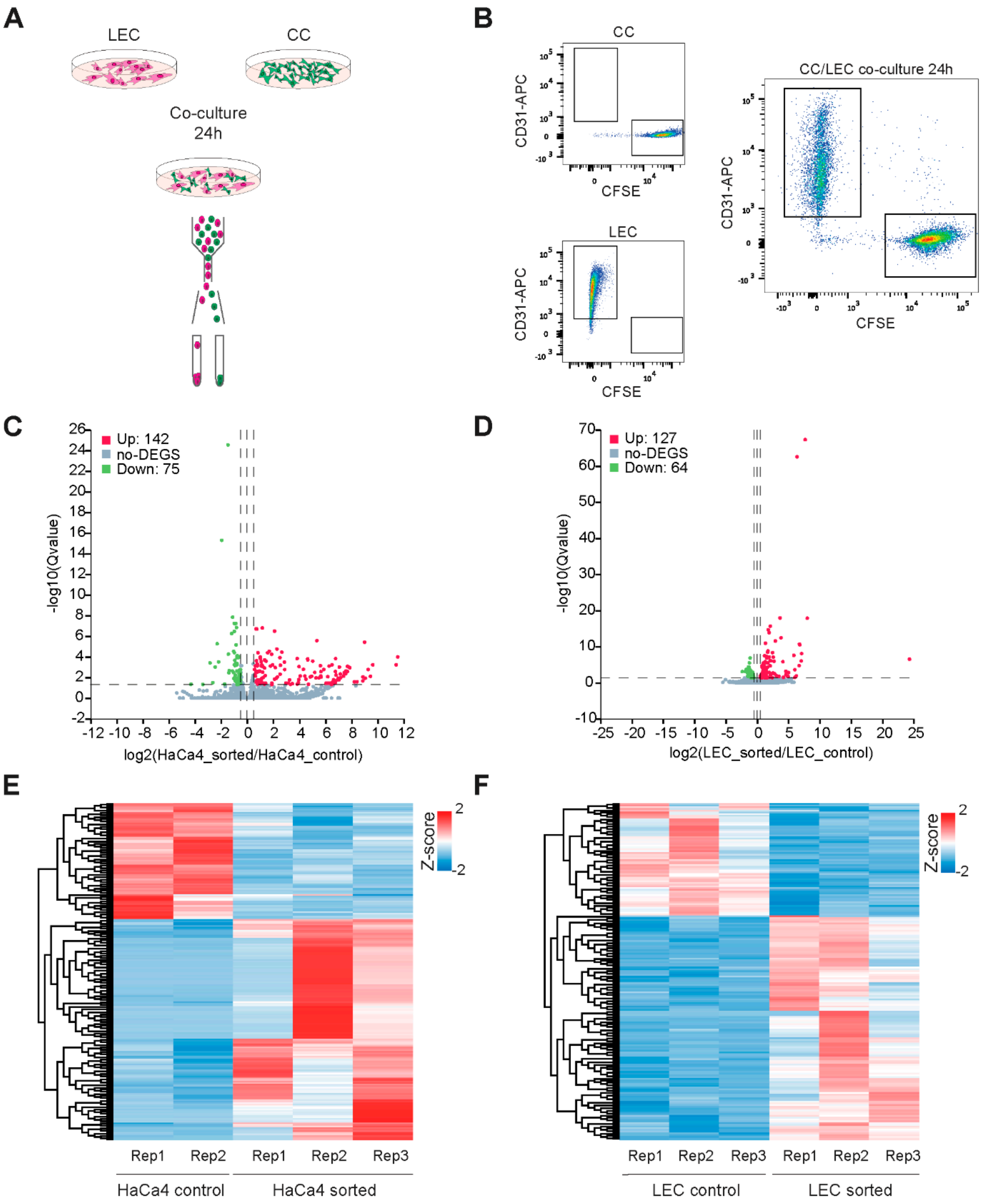

Figure S3. LEC and cancer cells gene expression profiling upon co-culture. (**A**) Schematic illustrating the experimental strategy. (**B**) Sorting plots, cancer cells (CC) and LEC were sorted according to the positivity to the CFSE membrane-permeable fluorescent dye and CD31 expression, respectively. (**C**) and (**D**) Volcano plots of differentially expressed genes in HaCa4-sorted vs. HaCa4 and LEC-sorted vs LEC. (**E**) and (**F**) Heat maps of differentially expressed genes in HaCa4-sorted vs. HaCa4 and LEC-sorted vs. LEC.

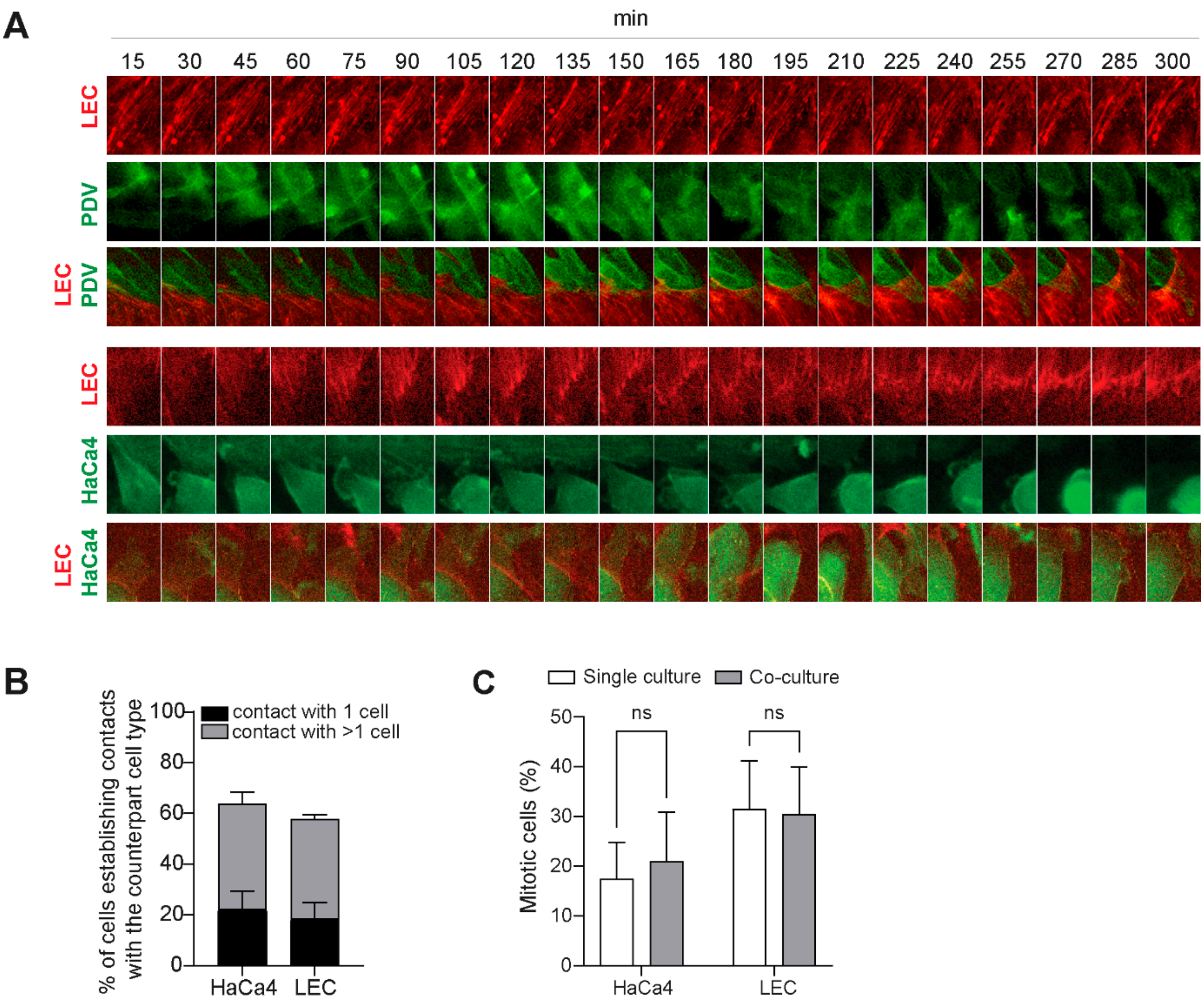

Figure S4. LEC and cancer cells interact dynamically over time, establishing heterotypic interactions without influencing their proliferation. (**A**) Kymograph showing the permanence of homotypic and heterotypic cell-cell contacts (15-300 min) between the analyzed cell types. (**B**) Percentage of HaCa4 cells establishing cell contacts with one or more LEC and vice versa. (**C**) Quantification of mitotic HaCa4 and LEC cells in single-culture and co-culture (n=4). n.s. Non-significant. $p \geq 0.05$.

Supplementary Videos

Video S1. PDV-LifeAct-GFP and LEC-LifeAct-RFP in co-culture interact dynamically, maintaining reciprocal connections over time. Cancer cells locate on top or bottom of the LEC forming dynamic ruffles and lamellipodia. Scale bar=50 μm.

Video S2. HaCa4-LifeAct-GFP and LEC-LifeAct-RFP in co-culture interact dynamically, maintaining reciprocal connections over time. Cancer cells locate on top or bottom of the LEC forming dynamic ruffles and lamellipodia. Scale bar=50 μm.

Supplementary Table

Table S1. List of primers used in the study.

Gene	Forward Primer (5' -> 3')	Reverse Primer (5' -> 3')
Hprt	GATCACTCAACGGGGGACATAAA	CTTGCGCTCATCTTAGGCTTTGT
Kdr	TTTGGCAAATACAACCCTTCAGA	GCAGAAGATACTGTCACCACC
Angpt2	TCGCTGGTGAAGAGTCCAAC	GTCAAACCACCAGCCTCCTG
Itga4	CCAGGCATTCATGCGGAAAG	ATGCCCAAGGTGGTATGTGG
Icam1	AGCCTCCGGACTTTCGATCT	TGTTTGTGCTCTCCTGGGTC
Lama4	CTCGGAAGCAAAAACGCCAA	GGAGCTCACAGGCTTGGAAT
Vcam1	CTTTATGTCAACGTTGCCCCC	AAATGCCGGAATCGTCCCTT
Itga7	CTGCTGTGGAAGCTGGGATTC	CTCCTCCTTGAACTGCTGTCG
